# Food security and pluriactivity in the Brazilian Amazon: a study on small rural establishments

**DOI:** 10.1590/0102-311XEN002924

**Published:** 2025-03-24

**Authors:** Graziela Gomes Bezerra, Alexandre Gori Maia, Elyson Ferreira de Souza

**Affiliations:** 1 Instituto de Economia, Universidade Estadual de Campinas, Campinas, Brasil.; 2 Departamento de Economia, Universidade Federal do Acre, Rio Branco, Brasil.

**Keywords:** Food Security, Rural Population, Community Development, Seguridad Alimentaria, Población Rural, Desarrollo Comunitario

## Abstract

The number and share of rural households whose members conduct non-agricultural activities has grown rapidly in the Amazon. This article analyzes the differences in food and nutrition security levels between small rural establishments dedicated exclusively to agricultural activities (non-pluriactive) versus those dedicated simultaneously to agricultural and non-agricultural activities (pluriactive) in the Amazon. The research uses data from the food security supplements of the *Brazilian National Household Sample Survey* (PNAD, acronym in Portuguese) for 2004, 2009 and 2013. The food security classification is based on the *Brazilian Food Insecurity Scale* (EBIA, acronym in Portuguese). A decomposition method was applied to quantify the differences in the occurrence of food security between the two groups of establishments attributed to their socioeconomic characteristics - such as income and education - and to unobservable factors - such as access to quality food. The results show higher food security levels for pluriactive establishments, with the main factors explaining this difference being associated with household head income and education. The final discussion demonstrates the importance of pluriactivity and rural development policies as strategies to promote food security in the Amazon.

## Abstract (es)

Hubo un rápido aumento de la participación de miembros de hogares rurales en actividades no agrícolas en la Amazonía. Este artículo analiza las diferencias en los niveles de seguridad alimentaria y nutricional entre los pequeños establecimientos rurales dedicados exclusivamente a la actividad agrícola (no pluriativos) frente a los que se dedican simultáneamente a actividades agrícolas y no agrícolas (pluriativos) en la Amazonía. Se utilizaron datos de los suplementos de seguridad alimentaria de la *Encuesta Nacional por Muestra de Domicilios* (PNAD, por su sigla en Portugués), para los años 2004, 2009 y 2013. La clasificación de seguridad alimentaria se basó en la *Escala Brasileña de Inseguridad Alimentaria* (EBIA). Se aplicó un método de descomposición para cuantificar las diferencias en la ocurrencia de la seguridad alimentaria entre los dos grupos de establecimientos en cuanto a características socioeconómicas, como ingresos y nivel educativo, y a factores no observables, como acceso a alimentos de calidad. Los resultados destacaron que los establecimientos pluriativos presentaron mayores niveles de seguridad alimentaria y que los principales factores que explican esta diferencia están asociados a ingresos y nivel educativo del responsable. Se destaca la importancia de las políticas de pluriactividad y desarrollo rural como estrategias para promover la seguridad alimentaria en la Amazonía.

## Introduction

This study analyzes food and nutrition security determinants among small rural establishments in the Brazilian Amazon, showing the differences between households whose members diversify income sources with agricultural and non-agricultural activities (pluriactive households) and households whose members are dedicated exclusively to agricultural activities (non-pluriactive households). The hypothesis to be analyzed is that pluriactive households would enjoy higher food and nutrition security by diversifying income sources ^1^ ^,^ ^2^, thereby ensuring regular and stable food supply for the household ^3^. In turn, income and food supply for households dedicated exclusively to agriculture would be subject to the instability and risks associated with agricultural production, credit availability, and access to consumer market ^4^ ^,^ ^5^ ^,^ ^6^. 

The Amazon provides a particular case to assess the impacts of pluriactivity on food and nutrition security. First, because the region is rich in natural resources that would favor activities associated with family farming, such as extractivism, artisanal fishing and subsistence agriculture ^7^. Throughout history, the region has undergone several economic cycles associated with native extractive products, such as rubber, cocoa, wood and chestnut ^8^. Currently, the region has a diverse food production for local consumption, such as rice, beans, corn, macaxeira, chestnut, in addition to livestock ^9^ ^,^ ^10^. This diversity enables the Amazon to develop a structure that favors food security by strengthening the trade of these products and the integration of producers into markets ^10^. 

Despite the abundant natural resources and diverse agricultural production, the Amazon Region has a low human development index (HDI) in relation to other Brazilian states ^11^. For example, in 2010, municipalities in the North Region, all in the Legal Amazon, had HDI comparable only to that of the Northeast Region (0.667 and 0.663, respectively) and 10 percentage points lower than that of the Southeast Region (0.766), which was the most developed region in terms of HDI. Food insecurity is also high and more severe in rural areas than in urban areas ^12^. In the North Region, in 2023, 38% of households in urban areas and 47% in rural areas reported food insecurity, against only 17% in urban areas and 14% in rural areas in the South Region, the least affected by food insecurity ^13^. 

In rural areas of the Amazon, there are at the same time traditional systems based on the collection of products from nature and modern agricultural systems with intensive use of technologies ^14^. Smallholder farmers are in a more vulnerable situation, since they compromise much of the low production and income for food consumption ^15^. The process of concentration and modernization of agricultural production in the region also puts pressure on the economic and social sustainability of small agricultural production, requiring investments to adopt new agricultural technologies and adapt to the new rules and requirements of the food market ^16^. A demographic aggravating factor for small family farming in the Amazon, which is still labor-intensive, is the process of young people - especially the most educated - shifting to urban economy activities ^17^. 

Family farming is responsible for a relevant share of food production in Brazil, especially food items that play an important role in the daily consumption of farmers themselves, such as cassava ^18^. However, few studies have analyzed the associations between family farming, its socioeconomic conditions, and food and nutritional security, especially in the Amazon. Studies in other regions indicate that the main reasons for family farming households having a high incidence of food insecurity would be low family income and low food variety ^19^.

If socioeconomic conditions are fundamental determinants of food security, then providing better income and employment opportunities in rural areas would be an important policy to promote food and nutrition security among smallholder farmers. Pluriactivity in rural households has been indicated as an alternative to diversify income and ensure sustainable smallholding farming and household food security. Pluriactivity has grown markedly in the Amazon region, with relevant impacts on income distribution ^20^. 

This study analyzes the determinants of differences in food and nutrition security level between pluriactive and non-pluriactive households in the rural Amazon. The study answers two main questions. First, what are the differences in food and nutritional security between pluriactive and non-pluriactive farmers? Second, what are the main factors that explain these differences: would they be socioeconomic characteristics (such as income and education) or unobservable factors (such as food supply instability)?

## Method

### Data source and variables

The study stacked the microdata database of the *Brazilian National Household Sample Survey* (PNAD, acronym in Portuguese) for 2004, 2009 and 2013, provided by the Brazilian Institute of Geography and Statistics (IBGE, acronym in Portuguese) ^21^. These years include the supplementary Brazilian food security research, in addition to a rich diversity of information about the labor market belonging to the basic questionnaire. This information include, we note, for this study, the data on the characteristics of the agricultural enterprises of employers and self-employed workers, including the size of the establishment, which are available until 2015. 

**Figure 1:**
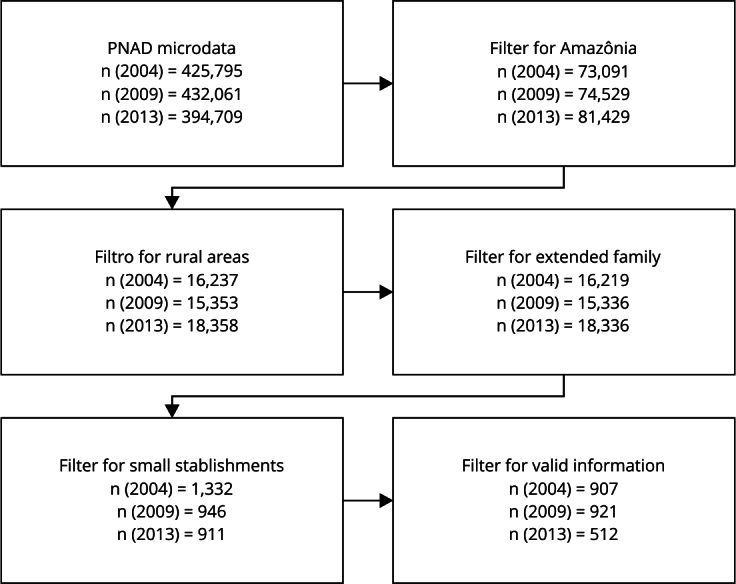
Size of the analysis sample (n) at each stage of data processing.

Figure 1 illustrates the steps of processing the database. First, we delimited the sample to the share of rural households located in the states of Acre, Amapá, Amazonas, Pará, Rondônia, Roraima, Tocantins, Maranhão and Mato Grosso. Although only a portion of the areas in the states of Tocantins, Maranhão and Mato Grosso belong to the Legal Amazon, we chose to consider, in a simplified way, all rural households in these states due to limitations in the territorial delimitation of the PNAD. It is important to emphasize that this approach does not introduce major distortions in the analyses, since most rural households in these states are in the Legal Amazon ^22^.

PNAD: *Brazilian National Household Sample Survey*.

Subsequently, we delimited the sample to households located in rural areas with employers or self-employed workers in agriculture. Finally, we delimited the portion composed of small farmers to reduce selectivity into pluriactive and non-pluriactive farmer groups. We defined a strategy to identify small establishments using the property area available in the PNAD. Brazilian *Law n. 11,326,* of July 24, 2006 ^23^, regulated by *Decree n. 9,064*, of May 31, 2017 ^24^, defines small family farmers as possessing up to four fiscal modules. The sizes of a fiscal module in the Amazon range between approximately 40 and 100 hectares. We restricted the analysis to properties with an area between 1 and 400 hectares to consider all properties that can be classified as small family farm. A similar strategy was adopted in other studies in the literature ^20^ ^,^ ^25^.

We excluded household residents characterized as domestic workers, relatives of domestic workers, and people who lived in the household on a pension basis from the sample to keep the focus on occupied extended families. After eliminating households with undeclared information for the variables considered in this study, the final sample had 907 observations for 2004, 921 observations for 2009, and 512 observations for 2013. To increase the statistical power of the analyses, we stacked the data for the three years, forming a database with 2,340 rural households.

We defined as pluriactive households those in which at least one member had agricultural activity and (at least) another member had non-agricultural activity. It is essential to emphasize that activities carried out in pluriactivity can occur inside or outside the family property ^5^. Non-pluriactive households are those whose members have exclusively agricultural activities. The sample contains 1,921 (82.1%) non-pluriactive households and 419 (17.9%) pluriactive households.

The food and nutriton secrurity classification is based on the *Brazilian Food Insecurity Scale* (EBIA), which is a psychometric scale that seeks to detect households’ perception of food availability ^26^. The EBIA classification is based on the sum of the affirmative answers of a questionnaire with 14 questions. Table 1 presents the scale with the sum of the affirmative answers to classify households into: food security, mild food insecurity, moderate food insecurity, and severe food insecurity. To simplify decomposition analyses (explained below), the last three food insecurity categories were clustered into a single category. This clustering is similar to that adopted in the IBGE releases ^12^. Thus, this work defines the binary variable *Y* that assumes *1* in the case of the household being in a food security condition and *0* in the case of food insecurity (mild, moderate or severe). The sample contains 1,324 households without food security (56.6%) and 1,016 (43.4%) households with food security.

**Table 1:** Food security classification according to *Brazilian Food Insecurity Scale* (EBIA) based on the *Brazilian National Household Sample Survey* (PNAD) Supplementary Food Security Survey Questionnaire.

Classification	Number of positive answers per household	
	With persons aged under 18 years	Without persons aged under 18 years
Food security	0	0
Mild food insecurity	1-5	1-3
Moderate food insecurity	6-9	4-5
Severe food insecurity	10-14	6-8

Source: PNAD, 2013 − Supplementary Food Security Survey Report ^21^.

The control variables include socioeconomic information that can simultaneously influence access to food and condition of pluriactivity: binary that assumes 1 if any member has activity for self-consumption (Self-consumption); (*log*) total area of the property (Total Area); (*log*) household income (Household Income − deflated using the Brazilian National Index of Prices for the Consumer [INPC, acronym in Portuguese] based on 2013); share of household income from non-work sources (Non-Work Income − such as pensions and income transfer programs); total residents aged over 18 years; total residents aged under 18 years; binary that assumes 1 when the household head is female (Woman); if the household head cohabits with a spouse (Cohabiting with Spouse); age of the household head (Age); years of education of the household head (Years of Schooling); nimary that assumes 1 if the household head is an employer (Employer); binary that assumes 1 if the household head is the owner of the establishment (Owner); if the household sells part of the agricultural production (Sells Production).

### Oaxaca-Blinder decomposition

We used the decomposition method based on the proposal of Oaxaca ^27^ and Blinder ^28^ to estimate the differences in food and nutrition security between non-pluriactive households (group A) and pluriactive households (group B) due to two components: (i) differences associated with the socioeconomic characteristics of the households (control variables); and (ii) unobserved factors associated, for example, with regularity and stability in the access and consumption of quality food. By quantifying the contribution of several observable and unobservable factors to the differences between the groups of pluriactive and non-pluriactive households, the method enables tracing the main sources of inequality in food and nutrition security in the rural Amazon. This method is considerably widespread in the socioeconomic literature to analyze inequalities in income distribution, and has recently also been adopted in studies of inequality in health care and food security ^29^ ^,^ ^30^.

The first step is to adjust the linear probability model (LPM) for the probability that the non-pluriactive household (subscribed *l = A*) or pluriactive household (*l = B*) presents food and nutrition security (*Y = 1*), given by:

$$P\left(Y_{l}=1\right)=x_{l}\beta_{l}$$ (1)

where the vector *x* contains the control variables and the *β* vector, their respective coefficients. We used LPM instead of nonlinear models (*logit* and *probit*) for the analytical simplicity of the linear decomposition (explained below) and for the fact that the marginal effects of these models on the mean values of the independent variables (which is the central interest of decomposition analysis) are usually similar. 

The difference between the ratios of non-pluriactive households (A) and pluriactive households (B) with food security is given by:

$$∆\bar{Y}={\bar{Y}}_{A}-{\bar{Y}}_{B}$$ (2)

By developing Equations (1) and (2) algebraically, we obtain ^31^:

$$∆\bar{Y}=\underset{explicado}{\underbrace{{\left({\bar{x}}_{A}-{\bar{x}}_{B}\right)}^{'}{\hat{\beta }}^{*}}}+\underset{inexplicado}{\underbrace{\underset{inexplicadoA}{\underbrace{{\bar{x}}_{A}^{'}\left({\hat{\beta }}_{A}-{\hat{\beta }}^{*}\right)}}+\underset{inexplicadoB}{\underbrace{{\bar{x}}_{B}^{'}\left({\hat{\beta }}^{*}-{\hat{\beta }}_{B}\right)}}}}$$ (3)

Where ${\bar{x}}_{A}$ and ${\bar{x}}_{B}$ are the mean values of the explanatory variables of the non-pluriactive and pluriactive households, respectively; ${\hat{\beta }}_{A}$ and ${\hat{\beta }}_{B}$ are the ordinary least squares estimates for the LPM (Equation 1) of the respective groups; and ${\hat{\beta }}^{*}$ is a mean of the estimates of the coefficients of the two groups. 

The first part of Equation (3) represents the portion of food and nutrition security difference due to the average socioeconomic characteristics of the non-pluriactive and pluriactive households. For example, the portion of food and nutition security difference due to the fact that pluriactive households have a higher average income or to the fact that their heads have a higher education level compared with non-pluriactive households. The second part represents the portion that is unexplained, or due to the differences between the coefficients of the models for non-pluriactive households (unexplained A) and pluriactive households (unexplained B) . For example, the portion of food and nutrition security difference due to the fact that pluriactive households have a greater positive effect of income on food security (coefficient in ${\hat{\beta }}_{B}$ ) compared with non-pluriactive households (coefficient in ${\hat{\beta }}_{A}$ ). This effect differentiated between the groups may be due to a number of factors, including unobserved factors, such as the higher food supply stability in households with the same income.

## Results

Table 2 presents descriptive statistics for non-pluriactive and pluriactive households. Food and nutrition security is more frequent among pluriactive households than among non-pluriactive households (52.3% versus 41.5%, difference of 10.8 percentage points). When compared with non-pluriactive households, pluriactive households also stand out for having better socioeconomic indicators, such as higher average income (21% higher), less dependence on other income sources (such as pensions and income transfer programs), more family members (young people and adults), and higher average education of household heads. 

**Table 2:** Descriptive statistics, Amazon, Brazil, 2004, 2009, and 2013.

Variables	Non-pluriactive		Pluriactive	
	Mean	SD	Mean	SD
Food security	0.415	0.493	0.523	0.500
Self-consumption	0.858	0.349	0.847	0.360
Total area	50.751	56.597	53.322	58.114
Household income	944.62	1,100.60	1,143.65	1,769.31
Non-work income	0.408	0.218	0.322	0.179
Total persons aged over 18 years	2.464	1.003	2.893	1.126
Total persons aged under 18 years	1.970	1.716	2.107	1.634
Woman	0.064	0.245	0.093	0.291
Cohabiting with spouse	0.881	0.324	0.931	0.254
Age	44.46	13.76	45.49	13.35
Years of schooling	3.274	3.220	3.885	3.516
Employer	0.054	0.226	0.074	0.262
Owner	0.790	0.408	0.835	0.371
Sells production	0.891	0.311	0.893	0.310

SD: standard deviation

Source: prepared by the authors with data from the Brazilian Institute of Geography and Statistics (IBGE) ^22^, *Brazilian National Household Sample Survey* (PNAD), 2004, 2009, 2013 ^21^.

Table 3 presents the LPM estimates for the probability of food and nutrition security in non-pluriactive and pluriactive households (Equation 1). The variables with significant effects simultaneously in the models for non-pluriactive and pluriactive households are household income and household head education. The two variables have a positive relation with food and nutrition security, that is, the higher the household income and household head education, the higher the probability that the household presents food and nutritional security. In the model for non-pluriactive households, the presence of a spouse in the household and the fact that the household head is an agricultural employer also present a positive and significant relation (p < 0.05) with food and nutritional security. In turn, in these same households, the prevalence of other income sources (such as income transfer programs) and activity for self-consumption present negative and significant effects on food and nutritional security. 

**Table 3:** Estimates of linear probability models for the binary dependent variable food security in non-pluriactive and pluriactive households, Amazônia, Brazil, 2004, 2009 and 2013.

Variables	Group A: non-pluriactive	Group B: pluriactive
Total area	0.006	0,002
	(0.010)	(0,023)
Household income	0,073 *	0.097 *
	(0.014)	(0.029)
Non-work income	-0.147 **	-0.153
	(0.055)	(0.152)
Total persons aged over 18 years	0.018	0.024
	(0.011)	(0.023)
Total persons aged under 18 years	0.005	0.019
	(0.007)	(0.016)
Woman	-0.005	-0.063
	(0.046)	(0.086)
Cohabiting with spouse	0.181 *	0.072
	(0.036)	(0.098)
Age	-0.002	-0.001
	(0.001)	(0.002)
Years of schooling	0.019 *	0.018 ***
	(0.004)	(0.008)
Employer	0.020	0.023
	(0.049)	(0.095)
Owner	0.070 ***	0.093
	(0.027)	(0.068)
Sells production	0.053	-0.028
	(0.036)	(0.079)
Self-consumption	-0.167 *	-0.012
	(0.032)	(0.068)

Source: prepared by the authors with data from the Brazilian Institute of Geography and Statistics (IBGE) ^22^, *Brazilian National Household Sample Survey* (PNAD), 2004, 2009, 2013 ^21^.

* p < 0.001;

** p < 0.01;

*** p < 0.05.

**Figure 2:**
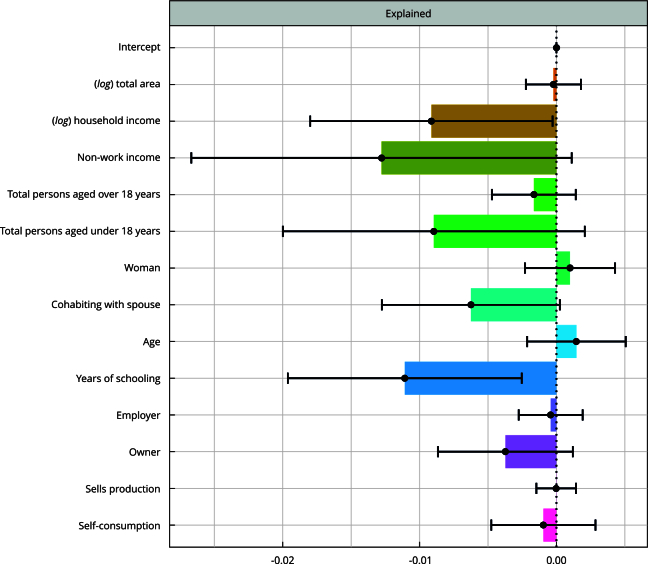
Food security prevalence difference between non-pluriactive and pluriactive households explained by the independent variables (with 95% confidence intervals), Amazon, Brazil, 2004, 2009 and 2013.

Figure 2 shows the estimates of the effects explained for each of the independent variables (first part of Equation 3). In general, the effects explained by the independent variables represent -5.3 percentage points of the total difference of -10.8 percentage points between the proportion of non-pluriactive and pluriactive households with food and nutritional security. That is, half of the food and nutritional security difference between the two groups is due to the fact that pluriactive families present socioeconomic characteristics that favor the incidence of food security. The other half of the difference is due to factors that are not explained, or not controlled by the model. Estimates for the unexplained effects associated with each variable are not presented because the interpretations are not intuitive.

The socioeconomic characteristics with the most significant contributions to explain the food and nutritional security difference between the groups are household income and household head education. Approximately one percentage point of the difference is due to the fact that pluriactive households have a higher income and another percentage point is due to the fact that heads of pluriactive households have higher education than those of non-pluriactive households. The higher prevalence of spouses cohabiting in pluriactive households also contributes marginally to explain this difference. 

## Discussion

The results of this article show that pluriactive rural households have a markedly higher prevalence of food and nutritional security than non-pluriactive households and that half of this difference is due to the better socioeconomic conditions of pluriactive households (such as income and education). 

Pluriactivity has been found as a phenomenon influencing the deactivation of rural establishments, since it would compromise the availability of labor for agricultural activity and the succession of the family unit ^32^ ^,^ ^33^. However, recent evidence indicates that pluriactivity plays an important role in diversifying and increasing income in rural households ^34^, a phenomenon that is reproduced in the Brazilian Amazon ^25^. This article presents further evidence of the importance of pluriactivity for rural development in Brazil, increasing food and nutritional security levels among small rural establishments in the Amazon. Family farming in the region is affected by demographic pressure (exodus of young people) and the recent advance of highly technology-intensive agriculture. Finding alternatives to improve the income level and food consumption of these families is essential to ensure social and economic sustainability in the region. 

Half of the difference between pluriactive and non-pluriactive households is due to the fact that pluriactive households have socioeconomic characteristics that favor food security. Among the characteristics analyzed, household head education and income contribute most significantly to increase the food and nutritional security of pluriactive households compared with non-pluriactive households. These results corroborate the literature on the importance of increasing income and education to reduce the high rates of food insecurity in the country ^35^. 

Poverty still affects a significant share of households in rural areas of the Brazilian Amazon ^11^. The higher income of pluriactive households reduces the budgetary constraint for purchasing food in sufficient quantity and quality for all family members. In turn, education is associated with access to information, employability and agricultural productivity, with direct impacts on poverty and food security in rural areas ^36^. Although pluriactive households have higher education levels than non-pluriactive households, the average education is still extremely low, not exceeding four years of schooling. In order to increase food and nutrition security levels in the region, it is essential, above all, to implement measures to promote access to quality education in the region. There are still substantial inequalities in access to and quality of education between regions in Brazil. For example, only 48% of elementary schools in the North Region have broadband internet access, compared to 92% in the Southeast Region ^37^. 

The study also found that half of the food and nutritional security differences between pluriactive and non-pluriactive households are due to unobservable factors. That is, they are not exclusively due to the better socioeconomic conditions of pluriactive households, such as higher income and higher levels of education. For example, increased income may not necessarily result in improved food and nutritional security levels if the family simultaneously needs to invest in means to secure or increase agricultural production, or even invest in access to basic rights such as health care and education. In a context of volatile agricultural product prices and increased climate risks, there is growing pressure for investments in modernization and reduction of the environmental impacts of agricultural production ^38^. Similarly, increasing access to information through higher levels of education may not necessarily result in increased family farming food production if, for example, there is difficulty in accessing credit for investment in agricultural production ^39^. Pluriactive households could circumvent these credit limitations with access to more diversified income sources, not exclusively associated with agriculture. 

It is also important to note that, despite the positive results found for pluriactivity in the Amazon, it would not be able, by itself, to secure the food and nutritional security of rural households. Food and nutrition insecurity is high in both groups of farmers. It is important to increase the productivity of family farming to increase both income and the availability of food for household consumption. The region is also marked by extreme social and territorial inequalities. For example, long distances and limitated means of transportation in the region hinder activities to be conducted far from home. Job creation programs in urban areas exclude the population that needs to travel long distances to access the workplace. 

Finally, we note some limitations of this research. First, the PNAD, despite providing a substantial range of information on the labor market and agricultural activities, has limited information on the access to, quantity and quality of food items consumed at home. Despite being an important source of information about food and nutritional security, the EBIA is a psychometric scale focused on the perception and experience of hunger. For example, because it concerns the resident’s perception of hunger, there may be results related to hidden hunger, which is the situation in which an individual does not always eat the necessary meals with sufficient quality and nutritional power to meet their needs ^40^.

## Conclusion

Even with difficulties as to data availability, this research presents relevant results, demonstrating how pluriactivity contributes to increase food and nutritional security in the rural Amazon. The growth of non-agricultural activities in the Amazon is a result of recent changes in rural society - such as greater communication and integration with urban areas driven by increased access to public transportation - and in the labor market - such as the growing autonomy of agricultural activities, leading to lower participation of family members ^34^. Pluriactivity enables farmers to combine agricultural or extractive activities with often more stable income sources, mitigating risks associated with agricultural activities, such as climate change, pests, and price fluctuations. Other studies in developing countries have also found that the existence of non-agricultural income in rural households has a positive impact on food expenditure, providing higher food diversity ^41^ ^,^ ^42^. However, pluriactivity cannot be considered as the only solution to the food and nutritional security issue in the rural Amazon, as food insecurity is high in both family groups. The low socioeconomic development in the region requires more complex rural development policies. For example, the persistent exodus of young people to urban areas requires the adoption of less labor-intensive agricultural strategies, for example through mechanization and adoption of agricultural technologies. This requires massive investments in education, rural extension and infrastructure to facilitate the adoption of new technologies and more sustainable practices ^14^. The assessment of how rural development policies in the rural Amazon would affect food and nutritional security would be an interesting development of this research.
