# Supplementary material for: Food security and pluriactivity in the Brazilian Amazon: a study on small rural establishments
Source: Cad Saude Publica. 2025 Mar 24;41(Suppl 1):e00002924. doi: 10.1590/0102-311XEN002924 (PMC11940860; doi:10.1590/0102-311XEN002924)
Supplement: Translation

# Translation: Segurança alimentar e pluriatividade na Amazônia brasileira: um estudo sobre pequenos estabelecimentos rurais

**DOI:** 10.1590/0102-311XPT002924

**Authors:** Graziela Gomes Bezerra, Alexandre Gori Maia, Elyson Ferreira de Souza

**Affiliations:** 1 Instituto de Economia, Universidade Estadual de Campinas, Campinas, Brasil.; 2 Departamento de Economia, Universidade Federal do Acre, Rio Branco, Brasil.

**Keywords:** Segurança Alimentar, População Rural, Desenvolvimento Comunitário

## Abstract

O número e a participação de domicílios rurais com membros praticando atividades não agrícolas tem crescido aceleradamente na Amazônia. Este artigo analisa as diferenças nos níveis de segurança alimentar e nutricional entre pequenos estabelecimentos rurais que se dedicam exclusivamente à atividade agrícola (não pluriativos) e aqueles que se dedicam simultaneamente a atividades agrícolas e não agrícolas (pluriativos) na Amazônia. A pesquisa utiliza dados dos suplementos de segurança alimentar da *Pesquisa Nacional por Amostra de Domicílios* (PNAD) dos anos de 2004, 2009 e 2013. A classificação da segurança alimentar baseia-se na *Escala Brasileira de Insegurança Alimentar* (EBIA). Aplica-se um método de decomposição para quantificar as diferenças na ocorrência de segurança alimentar entre os dois grupos de estabelecimentos atribuídas às suas características socioeconômicas, como renda e escolaridade, e a fatores não observáveis, como acesso a alimentos de qualidade. Os resultados destacam que os estabelecimentos pluriativos apresentam níveis mais elevados de segurança alimentar e que os principais fatores explicando essa diferença estão associados à renda e à escolaridade da pessoa responsável. A discussão final destaca a importância da pluriatividade e de políticas de desenvolvimento rural como estratégias para promover a segurança alimentar na Amazônia.

## Introdução

Este trabalho analisa os determinantes da segurança alimentar e nutricional entre pequenos estabelecimentos rurais na Amazônia brasileira, destacando as diferenças entre domicílios em que os membros diversificam a fonte de rendimentos com a prática a atividades agrícolas e não agrícolas (pluriativos) e os domicílios em que os membros se dedicam exclusivamente às atividades agrícolas (não pluriativos). A hipótese a ser analisada é a de que os domicílios pluriativos desfrutariam de uma maior segurança alimentar e nutricional por diversificar as fontes de rendimentos ^1^ ^,^ ^2^ e, assim, garantir uma provisão regular e estável de alimentos ^3^. Por sua vez, a renda e a provisão de alimentos nas propriedades dedicadas exclusivamente à agricultura estariam sujeitas à instabilidade e aos riscos associados à produção agrícola, à disponibilidade de crédito e ao acesso ao mercado de consumo ^4^ ^,^ ^5^ ^,^ ^6^.

A Amazônia oferece um caso particular para avaliar os impactos da pluriatividade sobre a segurança alimentar e nutricional. Primeiro, pelo fato de a região ser rica em recursos naturais que favoreceriam atividades associadas à agricultura familiar, como o extrativismo, a pesca artesanal e a agricultura de subsistência ^7^. Ao longo da história, a região passou por diversos ciclos econômicos vinculados a produtos nativos extrativos, como a seringueira, o cacau, a madeira e a castanha ^8^. A região atualmente apresenta uma diversa produção de alimentos orientada ao consumo local, como arroz, feijão, milho, macaxeira, castanha, além da pecuária ^9^ ^,^ ^10^. Essa diversidade permite que a Amazônia seja capaz de desenvolver uma estrutura que favoreça a segurança alimentar por meio do fortalecimento da comercialização desses produtos e da integração dos produtores nos mercados ^10^.

Apesar da riqueza em recursos naturais e a diversidade da produção agrícola, a Região Amazônica apresenta baixos índices de desenvolvimento humano (IDH) em relação aos demais estados brasileiros ^11^. Por exemplo, em 2010, o IDH dos municípios da Região Norte, todos inseridos na Amazônia Legal, era comparável apenas ao da Região Nordeste (0,667 e 0,663, respectivamente) e era dez pontos percentuais inferior ao da Região Sudeste (0,766), a região mais desenvolvida em termos de IDH. A insegurança alimentar também é elevada e mais grave nas áreas rurais que nas áreas urbanas ^12^. Na Região Norte, em 2023, 38% dos domicílios das áreas urbanas e 47% nas áreas rurais declararam insegurança alimentar, contra apenas 17% nas áreas urbanas e 14% nas áreas rurais da Região Sul, a menos afetada ^13^.

Nas áreas rurais da Amazônia, coexistem desde sistemas tradicionais baseados na coleta de produtos da natureza até sistemas agrícolas modernos com uso intensivo de tecnologias ^14^. Os pequenos produtores rurais estão em situação mais vulnerável, já que comprometem boa parte da baixa produção e do rendimento para o consumo de alimentos ^15^. O processo de concentração e modernização da produção agrícola na região também pressiona sua sustentabilidade econômica e social, exigindo investimentos para a adoção de novas tecnologias agrícolas e adaptação às novas regras e exigências do mercado de alimentos ^16^. Um agravante demográfico para a pequena produção familiar na Amazônia, que é ainda intensiva em mão de obra, é o processo de migração dos jovens, sobretudo os mais escolarizados, para as atividades da economia urbana ^17^.

A agricultura familiar é responsável por uma parcela relevante da produção de alimentos no Brasil, sobretudo de alimentos com função importante no consumo diário dos próprios agricultores, como a mandioca ^18^. Entretanto, são escassos os estudos analisando as associações entre a produção agrícola familiar, seus condicionantes socioeconômicos e a segurança alimentar e nutricional, sobretudo na Amazônia. Estudos em outras regiões indicam que os motivos centrais para as famílias de produtores apresentarem alta incidência de insegurança alimentar seriam a baixa renda familiar e a baixa variedade da alimentação ^19^.

Se as condições socioeconômicas são determinantes fundamentais da segurança alimentar, então prover melhores oportunidades de renda e emprego nas áreas rurais seria uma política importante para promover a segurança alimentar e nutricional entre pequenos produtores agrícolas. A prática da pluriatividade em domicílios rurais é apontada como uma alternativa para diversificar a renda e garantir a sustentabilidade da pequena produção e a segurança alimentar do domicílio. A pluriatividade tem crescido marcadamente na Região Amazônica, com impactos relevantes na distribuição de renda ^20^.

Este estudo analisa os determinantes das diferenças de grau de segurança alimentar e nutricional entre domicílios pluriativos e não pluriativos na Amazônia rural. O estudo responde a duas questões principais. Primeiro, quais as diferenças em segurança alimentar e nutricional entre produtores pluriativos e não pluriativos? Segundo, quais são os principais fatores que explicam essas diferenças: seriam as características socioeconômicas (como renda e escolaridade) ou fatores não observáveis (como instabilidade da provisão de alimentos)?

## Método

### Fonte de dados e variáveis

O estudo empilha a base de microdados da *Pesquisa Nacional por Amostra de Domicílios* (PNAD) dos anos 2004, 2009 e 2013, fornecida pelo Instituto Brasileiro de Geografia e Estatística (IBGE) ^21^. Esses anos contemplam a pesquisa suplementar de segurança alimentar brasileira, além de uma rica diversidade de informações sobre o mercado de trabalho pertencentes ao questionário básico. Entre essas informações, destacam-se, para este estudo, os dados sobre as características dos empreendimentos agrícolas de empregadores e trabalhadores por conta própria, incluindo o tamanho do estabelecimento, que estão disponíveis até o ano de 2015.

A Figura 1 ilustra as etapas de tratamento da base de dados. Primeiro, delimitamos a amostra à parcela de domicílios rurais localizados nos estados do Acre, Amapá, Amazonas, Pará, Rondônia e Roraima. Apesar de apenas uma parcela das áreas dos estados de Tocantins, Maranhão e Mato Grosso pertencerem à Amazônia Legal, optamos por considerar de forma simplificada todos os domicílios rurais desses estados por limitações da delimitação territorial da PNAD. É importante ressaltar que essa abordagem não introduz grandes distorções nas análises, uma vez que a maioria dos domicílios rurais desses estados está na Amazônia Legal ^22^.

**Figura 1:** Tamanho da amostra de análise (n) em cada estágio de tratamento dos dados.

Em seguida, delimitamos a amostra aos domicílios localizados nas áreas rurais com empregadores ou trabalhadores por conta própria na agricultura. Finalmente, delimitamos a parcela composta por pequenos produtores para reduzir a seletividade nos grupos de produtores pluriativos e não pluriativos. Definimos uma estratégia para identificar os pequenos estabelecimentos usando a área da propriedade disponível na PNAD. A *Lei nº 11.326*, de 24 de julho de 2006 ^23^, regulamentada pelo *Decreto nº 9.064*, de 31 de maio de 2017 ^24^, define pequenos produtores familiares como possuidores de até quatro módulos fiscais. As dimensões de um módulo fiscal na Amazônia variam entre aproximadamente 40 e 100 hectares. Restringimos a análise às propriedades com área entre 1 e 400 hectares para considerar todas as propriedades que podem se classificar como pequena produção familiar. Estratégia semelhante foi adotada em outros trabalhos da literatura ^20^ ^,^ ^25^.

Excluímos da amostra os residentes dos domicílios caracterizados como empregado doméstico, parente de empregado doméstico e as pessoas que viviam no domicílio em regime de pensionato, para manter o foco em famílias extensas ocupadas. Após eliminar os domicílios com informações não declaradas para as variáveis consideradas neste estudo, a amostra final apresentava 907 observações para o ano de 2004, 921 para 2009 e 512 observações para 2013. Para aumentar o poder estatístico das análises, empilhamos os dados dos três anos, formando uma base com 2.340 domicílios rurais.

Definimos como domicílios pluriativos aqueles em que pelo menos um membro praticava atividade agrícola e (pelo menos) outro membro exercia atividade não agrícola. É essencial ressaltar que as atividades realizadas na pluriatividade podem ocorrer dentro ou fora da propriedade familiar ^5^. Domicílios não pluriativos são aqueles em que os membros praticam exclusivamente atividades agrícolas. A amostra contém 1.921 (82,1%) domicílios não pluriativos e 419 (17,9%) domicílios pluriativos.

A classificação da segurança alimentar e nutricional baseia-se na *Escala Brasileira de Insegurança Alimentar* (EBIA), que é uma escala psicométrica que busca detectar a percepção dos domicílios sobre a disponibilidade de alimentos ^26^. A classificação da EBIA é baseada no somatório das respostas afirmativas de um questionário com 14 questões. A Tabela 1 apresenta a escala de soma das questões afirmativas para classificar os domicílios em: segurança alimentar, insegurança alimentar leve, insegurança alimentar moderada e insegurança alimentar grave. Para simplificar as análises da decomposição (explicadas abaixo), as três últimas categorias de insegurança alimentar foram agregadas em uma única categoria. Essa agregação é semelhante à adotada nas divulgações do IBGE ^12^. Assim, este trabalho define a variável binária *Y* que assume *1* no caso de o domicílio estar em condição de segurança alimentar e *0* no caso de insegurança alimentar (leve, moderada ou grave). A amostra contém 1.324 domicílios sem segurança alimentar (56,6%) e 1.016 (43,4%) domicílios com segurança alimentar.

**Tabela 1:** Classificação da segurança alimentar segundo a *Escala Brasileira de Insegurança Alimentar* (EBIA) com base no Questionário da Pesquisa Suplementar de Segurança Alimentar da *Pesquisa Nacional por Amostra de Domicílios* (PNAD), 2013.

Classificação	Quantidade de respostas afirmativas por domicílios	
	Com menores de 18 anos	Sem menores de 18 anos
Segurança alimentar	0	0
Insegurança alimentar leve	1-5	1-3
Insegurança alimentar moderada	6-9	4-5
Insegurança alimentar grave	10-14	6-8

Fonte: relatório da pesquisa suplementar de segurança alimentar da PNAD ^21^.

As variáveis de controle incluem informações socioeconômicas que podem simultaneamente influenciar o acesso alimentar e a condição de pluriatividade: binária que assume 1 se algum integrante pratica atividade para o autoconsumo (Autoconsumo), (*log*) área total da propriedade (Área Total), (*log*) renda domiciliar (Renda Domiciliar − deflacionada utilizando o Índice Nacional de Preços ao Consumidor [INPC], tendo como base o ano de 2013), proporção da renda domiciliar proveniente de fontes não relacionadas ao trabalho (Renda não Trabalho − como aposentadorias e programas de transferências de renda), total de moradores maiores de 18 anos, total de moradores menores de 18 anos, binária que assume 1 quando a pessoa responsável pelo domicílio for mulher (Mulher), se a pessoa responsável pelo domicílio vive com o cônjuge (Vive com o Cônjuge), idade da pessoa responsável (Idade), anos de estudo da pessoa responsável (Anos de Estudos), binária que assume 1 se a pessoa responsável é empregadora (Empregador), binária que assume 1 se é proprietária do estabelecimento (Proprietário), e se o domicílio vende parcela da produção agrícola (Vende a Produção).

### Decomposição de Oaxaca-Blinder

Utilizamos o método de decomposição baseado na proposta de Oaxaca ^27^ e Blinder ^28^ para estimar as diferenças de segurança alimentar e nutricional entre domicílios não pluriativos (grupo A) e pluriativos (grupo B) devido a dois componentes: (1) diferenças associadas às características socioeconômicas dos domicílios (variáveis de controle); e (2) fatores não observados associados, por exemplo, à regularidade e estabilidade no acesso e consumo de alimentos de qualidade. Ao quantificar a contribuição de vários fatores observáveis e não observáveis para as diferenças entre os grupos de domicílios pluriativos e não pluriativos, o método permite identificar as principais fontes de desigualdade na segurança alimentar e nutricional na Amazônia rural. Esse método é bastante difundido na literatura socioeconômica para analisar as desigualdades na distribuição de rendimentos e, recentemente, é também adotado em estudos de saúde e segurança alimentar desiguais ^29^ ^,^ ^30^.

O primeiro passo é ajustar o modelo de probabilidade linear (MPL) para a probabilidade de o domicílio não pluriativo (subscrito *l = A*) ou pluriativo (*l = B*) apresentar segurança alimentar e nutricional (*Y = 1*), dado por:

$$P\left(Y_{l}=1\right)=x_{l}\beta_{l}$$ (1)

em que o vetor *x* contém as variáveis de controle e o vetor *β*, os seus respectivos coeficientes. Utilizamos MPL em vez de modelos não lineares (*logit* e *probit*) pela simplicidade analítica da decomposição linear (explicada a seguir) e pelo fato de os efeitos marginais desses modelos sobre os valores médios das variáveis independentes (que é o interesse central da análise de decomposição) serem usualmente semelhantes.

A diferença entre as proporções de domicílios não pluriativos (A) e pluriativos (B) com segurança alimentar é dada por:

$$∆\bar{Y}={\bar{Y}}_{A}-{\bar{Y}}_{B}$$ (2)

Desenvolvendo algebricamente as Equações 1 e 2, obtém-se ^31^:

$$∆\bar{Y}=\underset{explained}{\underbrace{{\left({\bar{x}}_{A}-{\bar{x}}_{B}\right)}^{'}{\hat{\beta }}^{*}}}+\underset{unexplained}{\underbrace{\underset{unexplainedA}{\underbrace{{\bar{x}}_{A}^{'}\left({\hat{\beta }}_{A}-{\hat{\beta }}^{*}\right)}}+\underset{unexplainedB}{\underbrace{{\bar{x}}_{B}^{'}\left({\hat{\beta }}^{*}-{\hat{\beta }}_{B}\right)}}}}$$ (3)

Em que ${\bar{x}}_{A}$ e ${\bar{x}}_{B}$ são os valores médios das variáveis explicativas dos domicílios não pluriativos e pluriativos, respectivamente; ${\hat{\beta }}_{A}$ e ${\hat{\beta }}_{B}$ são as estimativas de mínimos quadrados ordinários para o MPL (Equação 1) dos respectivos grupos; e ${\hat{\beta }}^{*}$ é uma média das estimativas dos coeficientes dos dois grupos.

A primeira parte da Equação 3 representa a parcela da diferença de segurança alimentar e nutricional devido às características socioeconômicas médias dos domicílios não pluriativos e pluriativos. Por exemplo, a parcela da diferença de segurança alimentar e nutricional em função do fato de os domicílios pluriativos apresentarem uma renda média mais elevada ou do fato de seus responsáveis terem escolaridade mais elevada que nos domicílios não pluriativos. A segunda parte representa a parcela inexplicada, ou devida às diferenças entre os coeficientes dos modelos para domicílios não pluriativos (inexplicado A) e pluriativos (inexplicado B). Por exemplo, a parcela da diferença de segurança alimentar e nutricional em razão do fato de os domicílios pluriativos apresentarem um efeito positivo maior da renda sobre a segurança alimentar (coeficiente em ${\hat{\beta }}_{B}$ ) em comparação aos domicílios não pluriativos (coeficiente em ${\hat{\beta }}_{A}$ ). Esse efeito diferenciado entre os grupos pode estar relacionado a uma série de fatores, incluindo fatores não observados, como a maior estabilidade da provisão de alimentos em domicílios com a mesma renda.

## Resultados

A Tabela 2 apresenta as estatísticas descritivas para os domicílios não pluriativos e pluriativos. A segurança alimentar e nutricional é mais frequente entre os domicílios pluriativos do que entre os domicílios não pluriativos (52,3% contra 41,5%, diferença de 10,8 pontos percentuais). Os domicílios pluriativos também se destacam por apresentar melhores indicadores socioeconômicos em comparação aos domicílios não pluriativos, como maior renda média (21% superior), menor dependência de outras fontes de rendimento (como aposentadorias e transferências de renda), mais integrantes familiares (jovens e adultos) e escolaridade média mais elevada dos responsáveis.

**Tabela 2:** Estatísticas descritivas, Amazônia, Brasil, 2004, 2009 e 2013.

Variáveis	Não pluriativos		Pluriativos	
	Média	DP	Média	DP
Segurança alimentar	0,415	0,493	0,523	0,500
Autoconsumo	0,858	0,349	0,847	0,360
Área total	50,751	56,597	53,322	58,114
Renda domiciliar	944,62	1.100,60	1.143,65	1.769,31
Renda não trabalho	0,408	0,218	0,322	0,179
Total de maiores de 18 anos	2,464	1,003	2,893	1,126
Total de menores de 18 anos	1,970	1,716	2,107	1,634
Mulher	0,064	0,245	0,093	0,291
Vive cônjuge	0,881	0,324	0,931	0,254
Idade	44,46	13,76	45,49	13,35
Anos de estudo	3,274	3,220	3,885	3,516
Empregador	0,054	0,226	0,074	0,262
Proprietário	0,790	0,408	0,835	0,371
Vende a produção	0,891	0,311	0,893	0,310

DP: desvio padrão.

Fonte: elaborada pelos autores com os dados do Instituto Brasileiro de Geografia e Estatística (IBGE) ^22^, *Pesquisa Nacional por Amostras de Domicílios* (PNAD), 2004, 2009, 2013 ^21^.

A Tabela 3 apresenta as estimativas dos MPL para a probabilidade de segurança alimentar e nutricional nos domicílios não pluriativos e pluriativos (Equação 1). As variáveis com efeitos significativos simultaneamente nos modelos para não pluriativos e pluriativos são a renda domiciliar e a escolaridade da pessoa responsável. As duas variáveis apresentam relação positiva com a segurança alimentar e nutricional, ou seja, quanto maior a renda domiciliar e a escolaridade da pessoa responsável pelo domicílio, maior a chance de o domicílio apresentar segurança alimentar e nutricional. No modelo para domicílios não pluriativos, a presença de cônjuge no domicílio e o fato de a pessoa responsável ser empregadora agrícola também apresentam relação positiva e significativa (p < 0,05) com a segurança alimentar e nutricional. Por sua vez, nesses mesmos domicílios, a prevalência de outras fontes de rendimento (como transferências de renda) e a prática da atividade para o autoconsumo apresentam efeitos negativos e significativos sobre a segurança alimentar e nutricional.

**Tabela 3:** Estimativas dos modelos de probabilidade linear para a variável dependente binária segurança alimentar em domicílios não pluriativos e pluriativos, Amazônia, Brasil, 2004, 2009 e 2013.

Variáveis	Grupo A: não pluriativo	Grupo B: pluriativo
Área total	0,006	0,002
	(0,010)	(0,023)
Renda domiciliar	0,073 *	0,097 *
	(0,014)	(0,029)
Renda não trabalho	-0,147 **	-0,153
	(0,055)	(0,152)
Total de maiores de 18 anos	0,018	0,024
	(0,011)	(0,023)
Total de menores de 18 anos	0,005	0,019
	(0,007)	(0,016)
Mulher	-0,005	-0,063
	(0,046)	(0,086)
Vive com o cônjuge	0,181 *	0,072
	(0,036)	(0,098)
Idade	-0,002	-0,001
	(0,001)	(0,002)
Anos de estudo	0,019 *	0,018 ***
	(0,004)	(0,008)
Empregador	0,020	0,023
	(0,049)	(0,095)
Proprietário	0,070 ***	0,093
	(0,027)	(0,068)
Vende a produção	0,053	-0,028
	(0,036)	(0,079)
Autoconsumo	-0,167 *	-0,012
	(0,032)	(0,068)

Fonte: elaborado pelos autores com base nos dados do Instituto Brasileiro de Geografia e Estatística (IBGE) ^22^, *Pesquisa Nacional por Amostras de Domicílios* (PNAD), 2004, 2009, 2013 ^21^.

* p < 0,001;

** p < 0,01;

*** p < 0,05.

A Figura 2 apresenta as estimativas dos efeitos explicados para cada uma das variáveis independentes (primeira parte da Equação 3). De maneira geral, esses efeitos respondem por -5,3 pontos percentuais da diferença total de -10,8 pontos percentuais entre a proporção de domicílios não pluriativos e pluriativos com segurança alimentar e nutricional. Ou seja, metade da diferença de segurança alimentar e nutricional entre os dois grupos se deve ao fato de as famílias pluriativas apresentarem características socioeconômicas que favorecem a incidência de segurança alimentar. A outra metade da diferença se deve a fatores não explicados, ou não controlados pelo modelo. As estimativas para os efeitos inexplicados associados a cada variável não são apresentadas pelo fato de as interpretações não serem intuitivas.

**Figura 2:** Diferença da prevalência de segurança alimentar entre domicílios não pluriativos e pluriativos explicados pelas variáveis independentes (com intervalos de 95% de confiança), Amazônia, Brasil, 2004, 2009 e 2013.

As características socioeconômicas com as contribuições mais significativas para explicar a diferença de segurança alimentar e nutricional entre os grupos são a renda domiciliar e a escolaridade da pessoa responsável. Aproximadamente um ponto percentual da diferença se deve ao fato de os domicílios pluriativos apresentarem uma renda mais elevada, e outro ponto percentual, ao fato de os responsáveis pelos domicílios pluriativos terem escolaridade mais elevada que os de domicílios não pluriativos. A maior prevalência de cônjuge nos domicílios pluriativos também contribui marginalmente para explicar essa diferença.

## Discussão

Os resultados destacam que os domicílios rurais pluriativos apresentam uma prevalência marcadamente superior de segurança alimentar e nutricional que os domicílios não pluriativos e que metade dessa diferença se deve às melhores condições socioeconômicas nesses domicílios (como renda e escolaridade).

A pluriatividade já foi vista como um fenômeno influenciador da desativação do estabelecimento rural, já que comprometeria a disponibilidade de trabalho para a atividade agrícola e a sucessão da unidade familiar ^32^ ^,^ ^33^. Entretanto, evidências recentes indicam que a pluriatividade cumpre um papel importante na diversificação e aumento da renda nos domicílios rurais ^34^, fenômeno que se reproduz na Amazônia brasileira ^25^. Este artigo apresenta mais uma evidência da importância da pluriatividade para o desenvolvimento rural no Brasil, aumentando os níveis de segurança alimentar e nutricional entre pequenos estabelecimentos rurais da Amazônia. A agricultura familiar na região sofre com a pressão demográfica (êxodo de jovens) e o recente avanço da agricultura altamente intensiva em tecnologia. Encontrar alternativas para melhorar o padrão de renda e consumo alimentar dessas famílias é fundamental para garantir a sustentabilidade social e econômica na região.

Metade da diferença entre os domicílios pluriativos e não pluriativos se deve ao fato de os domicílios pluriativos apresentarem características socioeconômicas que favorecem a segurança alimentar. Entre as características analisadas, a renda e os anos de estudos da pessoa responsável são as que contribuem mais significativamente para aumentar a segurança alimentar e nutricional dos domicílios pluriativos em comparação aos não pluriativos. Esses resultados corroboram a literatura sobre a importância do aumento da renda e da escolaridade para reduzir os elevados índices de insegurança alimentar no país ^35^.

A pobreza ainda aflige uma parcela expressiva dos domicílios nas áreas rurais da Amazônia brasileira ^11^. A renda mais elevada dos domicílios pluriativos reduz a restrição orçamentária para aquisição de alimentos em quantidade e qualidade suficientes para todos os membros da família. Por sua vez, a escolaridade está associada ao acesso à informação, à empregabilidade e à produtividade agrícola, com impactos diretos sobre a pobreza e a segurança alimentar nas áreas rurais ^36^. Apesar de os níveis de escolaridade nos domicílios pluriativos serem superiores aos dos domicílios não pluriativos, a escolaridade média ainda é extremamente baixa, não ultrapassando quatros anos de estudos. Para aumentar a segurança alimentar e nutricional na região, é fundamental, antes de mais nada, promover medidas de promoção de acesso à educação de qualidade na região. Há ainda desigualdades marcantes no acesso e na qualidade do ensino entre as regiões no Brasil. Por exemplo, apenas 48% das escolas do Ensino Fundamental na Região Norte têm acesso à internet de banda larga, contra 92% na Região Sudeste ^37^.

O trabalho também destaca o fato de metade das diferenças de segurança alimentar e nutricional entre domicílios pluriativos e não pluriativos ser devida a fatores não observáveis. Ou seja, não se devem exclusivamente às melhores condições socioeconômicas dos domicílios pluriativos, como maior renda e maiores níveis de escolaridade. Por exemplo, o aumento da renda pode não necessariamente refletir em melhores níveis de segurança alimentar e nutricional se a família precisar simultaneamente investir em meios para garantir ou aumentar a produção agrícola, ou mesmo investir no acesso a direitos básicos, como saúde e educação. Em um cenário de volatilidade do preço dos produtos agrícolas e aumento dos riscos climáticos, há uma crescente pressão para investimentos em modernização e redução dos impactos ambientais da produção agrícola ^38^. Analogamente, aumentar o acesso à informação mediante níveis mais elevados de escolaridade pode não necessariamente se refletir no aumento da produção domiciliar de alimentos se, por exemplo, houver dificuldade de acesso ao crédito para investimento na produção agrícola ^39^. Os domicílios pluriativos poderiam contornar essas limitações de crédito com o acesso a uma fonte mais diversificada de renda, não associada exclusivamente à agricultura.

Também é importante destacar que, apesar dos resultados positivos apresentados para a pluriatividade na Amazônia, ela não seria capaz, por si só, de garantir a segurança alimentar e nutricional dos domicílios rurais. A insegurança é elevada nos dois grupos de produtores. É fundamental aumentar a produtividade da agricultura familiar para ampliar tanto a renda como a disponibilidade de alimentos para o consumo no domicílio. A região também é marcada por extremas desigualdades sociais e territoriais. Por exemplo, as longas distâncias e as limitações dos meios de transporte na região dificultam a realização de atividades fora do domicílio de moradia. Programas de geração de emprego nas áreas urbanas excluem a população que precisa deslocar longas distâncias para acessar o local de trabalho.

Por fim, destacamos algumas limitações desta pesquisa. Primeiro, a PNAD, apesar de apresentar uma rica gama de informações sobre o mercado de trabalho e atividades agrícolas, é limitada quanto ao acesso, à quantidade e à qualidade dos alimentos consumidos no domicílio. Apesar de ser uma fonte importante de informação sobre a segurança alimentar e nutricional, a EBIA é uma escala psicométrica que foca na percepção e experiência com a fome. Por exemplo, por ser a percepção do morador em relação à fome, podem ocorrer resultados relacionados à fome oculta, que é a situação em que um indivíduo nem sempre realiza as refeições necessárias com qualidade e poder nutritivo suficiente para suprir suas necessidades ^40^.

## Conclusão

Mesmo com as dificuldades encontradas na disponibilidade de dados, esta pesquisa apresenta resultados relevantes ao demonstrar como a pluriatividade contribui para aumentar a segurança alimentar e nutricional na Amazônia rural. O crescimento de atividades não agrícolas na Amazônia é reflexo das alterações recentes na sociedade rural, como a maior comunicação e integração com as áreas urbanas impulsionadas pelo aumento do acesso ao transporte público, e no mercado de trabalho, como a crescente autonomia das atividades agrícolas resultando na menor participação dos membros familiares ^34^. A pluriatividade permite que produtores combinem atividades agrícolas ou extrativistas com fontes muitas vezes mais estáveis de renda, mitigando os riscos associados às atividades agrícolas, como mudanças climáticas, pragas e flutuações de preços. Outros estudos em países em desenvolvimento também destacaram que a existência de rendimento não agrícola do domicílio rural impacta positivamente nas despesas alimentares, permitindo uma maior diversidade alimentar ^41^ ^,^ ^42^. Mas a pluriatividade não pode ser vista como solução única para o problema da segurança alimentar e nutricional na Amazônia rural, pois a insegurança alimentar é elevada nos dois grupos familiares. O baixo desenvolvimento socioeconômico na região exige políticas mais complexas de desenvolvimento rural. Por exemplo, o êxodo persistente de jovens para as áreas urbanas exige a adoção de estratégias agrícolas menos intensivas em trabalho; por exemplo, por meio da mecanização e da aplicação de tecnologias agrícolas. Para que isso se efetive, são necessários investimentos massivos em educação, extensão rural e infraestrutura que facilite a adoção de novas tecnologias e de práticas mais sustentáveis ^14^. A avaliação de como políticas de desenvolvimento rural na Amazônia afetariam a segurança alimentar e nutricional seria um desdobramento interessante desta pesquisa.
